# Evaluating the Connect with Pharmacy web-based intervention to reduce hospital readmission for older people

**DOI:** 10.1007/s11096-019-00887-3

**Published:** 2019-08-07

**Authors:** Fatima R. N. Sabir, Justine Tomlinson, Barry Strickland-Hodge, Heather Smith

**Affiliations:** 1 Medicines Management and Pharmacy Services, St James University Hospital, Leeds Teaching Hospitals NHS Trust, Beckett Street, Leeds, West Yorkshire LS9 7TF UK; 2 School of Healthcare, University of Leeds, Leeds, UK; 3 School of Pharmacy and Medical Sciences, University of Bradford, Bradford, UK

**Keywords:** Care transitions, Community pharmacy, Elderly, Hospital readmissions, Older people, United kingdom

## Abstract

*Background* The patient transition from a hospital to a post-discharge healthcare setting has potential to disrupt continuity of medication management and increase the risk of harm. “Connect with Pharmacy” is a new electronic web-based transfer of care initiative employed by Leeds Teaching Hospitals NHS Trust. This allows the sharing of discharge information between the hospital and a patient’s chosen community pharmacy. *Objective* We investigated whether the timely sharing of discharge information with community pharmacies via “Connect with Pharmacy” reduced hospital readmission rates in older patients. *Method* To evaluate intervention efficacy, hospital admission data was retrospectively collected. For primary analysis, admission rates were tracked 6-months prior (baseline) and 6-months post-intervention. Secondary measures included effect on total length of stay if readmitted, emergency department attendance and duration, and impact of polypharmacy. *Main outcome measure* The rate of non-elective hospital readmissions, 6-months post-intervention. *Results* In the sample (n = 627 patients; Mean age = 81 years), emergency readmission rates following the intervention (M = 1.1, 95% CI [0.98, 1.22]) reduced by 16.16% relative to baseline (M = 1.31, 95% CI [1.21, 1.42]) (W = 54,725; *p* < 0.001). There was no reduction in total length of stay. Subsidiary analysis revealed a post-intervention reduction in number of days spent in hospital lasting more than three days (χ^2^ = 13.37, df = 1, *p* < 0 .001). There were no statistically reliable differences in the remaining secondary measures. *Conclusion* The results showed a reduction in readmissions and potential post-intervention length of stay, indicating there may be further benefits for our older patients’ experiences and hospital flow.

## Impacts on practice

- The sharing of hospital discharge information with community pharmacies has the potential to reduce readmission rates in older people.
- Patients who are readmitted for more than 3 days following a timely sharing of hospital discharge information may have their stay shortened.
- Further study is needed to ascertain the full benefit of sharing hospital discharge information with community pharmacies.

## Introduction

There is growing evidence that deficiencies in quality of communication exist for patients transitioning between different care settings [1]. Information transfer between healthcare providers to support transitions has traditionally been restricted to post, fax or email, often accompanied by telephone calls [2–5]. These methods have led to problematic issues such as the omission of critical information within referral notes, individuals having difficulties reading handwritten discharge summaries and a lack of audit trail [2, 4, 6, 7]. Such problems, particularly when they involve medications in the early post-discharge period, may have substantial implications for patient safety [8–10], indeed a study has shown an association between discrepancies in discharge medication and readmission [11].

During hospital stay, nearly every patient will experience at least one modification in their medication regimen, and more than 75% of patients will have three or more changes [12]. Approximately 28–40% of medications are stopped within hospital and 45% of medicines prescribed at discharge are new [13]. Older people are particularly affected by these modifications as this population is prone to the complexities of multi-morbidities and polypharmacy, hence the need for greater post-discharge continuity of medication management [3, 14, 15]. Less than 10% of older patients will be discharged on the same medication they were admitted with and unsurprisingly, there is a rising level of unmet medication support needs for this high-risk population in the community.

Recent research indicates that 37% of older patients experience medication-related harm within 8 weeks following discharge and the annual financial burden to the UK National Health Service (NHS) is estimated to be £396m [8]. Prescribing errors and adverse drug events appear to increase as a function of the number of medications prescribed [16], with ten or more medications conveying a higher risk than four to nine [7]. Older patients also report a lack of understanding about their changed medication regimens which can lead to poor adherence, confusion and anxiety [17–19]. These post-discharge medicines-related problems may be addressed with more routine involvement of a community pharmacist [9, 20]. A recent systematic review found that when the hospital shared discharge information with community pharmacies there was a reduction in post-discharge medication discrepancies [20]. Other benefits of electronically transferring discharge information to and referring patients for support from their community pharmacies include: (1) quick and secure transfer of patient data; (2) reduced rate of readmissions; (3) reduced Emergency Department (ED) attendance; (4) shorter length of hospital stay for subsequent visits through improved medicines reconciliation; (5) reduced post-discharge medication errors [21–24]; (6) increased New Medicine Service consultations, which have shown to improve medication adherence by 10% [25]; and (7) increased post-discharge Medicine Use Reviews. These reviews have demonstrated that for every £1 spent by the health economy delivering the scheme, £3 of NHS spending on ED attendances, hospital admissions and medicines wastage is avoided [26].

“Connect with Pharmacy” (CwP) [27] is a new transfer of care initiative employed by Leeds Teaching Hospitals NHS Trust (LTHT), UK, to address these issues. It uses a secure web-based system (PharmOutcomes® provided by Pinnacle Health Partnership LLP) that allows timely electronic transfer of a patient’s discharge and handover information (including referrals for community pharmacy services) from the hospital to the patient’s chosen community pharmacy. The concept is based on a similar model employed at Newcastle-upon-Tyne NHS Foundation Trust [24]. CwP launched on 16th of January 2017 in collaboration with the Local Pharmaceutical Committee, Community Pharmacy West Yorkshire. Community Pharmacy West Yorkshire represents local community pharmacies in the area surrounding LTHT and is responsible for advancing the enhanced role of community pharmacy. The first phase of the CwP initiative focussed on patients using a compliance aid, with a plan to roll out the programme to all patients that could benefit from a further review by a community pharmacist post-discharge.

## Aims of the study

The primary aim of this study was to evaluate whether CwP reduced hospital readmission in older patients in the first phase of this programme (i.e. patients using a compliance aid). The secondary aim of the study was to identify if the intervention reduced ED attendances and if there was any effect on length of stay (LoS) of those patients who were readmitted following CwP referral, as well as assessing if the intervention had a greater effect for those patients taking multiple medications.

## Ethics approval

Ethics approval was granted by the Chair of the Biomedical, Natural, Physical and Health Sciences Research Ethics Panel at the University of Bradford on the 8th November 2017.

## Method

LTHT Medicines Management and Pharmacy Services purchased the PharmOutcomes® software. Information technology support was provided to implement the system and with any issues encountered, as well as liaising with LTHT Information Governance team to ensure processes met appropriate standards. An implementation team was set up by a Consultant Pharmacist (HS). The implementation team was instrumental in making sure that the project worked in practice, engaging with staff and regularly feeding back issues that could be resolved. Community Pharmacy West Yorkshire implemented the project in community pharmacies in Leeds and was responsible for providing training and feedback.

The CwP intervention process began at the point of admission to hospital and was carried out by a trained hospital pharmacy staff member. Information resources were developed to ensure that patients were fully informed about CwP and the benefits. This included a patient information leaflet and a web-page. Patients were asked for verbal consent before the CwP intervention took place. If the patient consented, or if the pharmacy staff member thought it was in their best interest (if the patient lacked capacity), the patient was registered on PharmOutcomes® and the community pharmacy would be notified of the patient’s admission to hospital. At the point of discharge, hospital pharmacy staff completed the CwP discharge information, including details of onward support required, and a copy of the discharge note was sent securely to the patient’s chosen community pharmacy.

Community pharmacies received a notification for each discharge note received from the hospital and were presented with a choice of either ‘accepting’ or rejecting’ it. If the community pharmacy did not acknowledge the referral in PharmOutcomes®, or did not carry out any action following receipt, the status of that referral remained recorded as ‘referred’, indicating no action had been taken. Data regarding community pharmacy activity was captured through PharmOutcomes®.

The present study adopted a retrospective quasi-experimental repeated measures design, in which all admissions pre-intervention were compared with admissions post-intervention in the same sample. There was no randomisation as no independent control arm was included. For patients that received the intervention during the 3-month period January to April 2017 (inclusive) hospital information was extracted to allow the identification of total admissions 6 months pre- and post-intervention. Extracted data included the patient’s age, gender, number of medicines at discharge, number of ED attendances and ED length of visit (hours) as well as ward admissions and average LoS (days). Elective admissions were excluded from analysis. If a patient was first assessed in ED and then transferred to a ward, this counted as a ward admission only. Before analysis, anomalies such as duplicated patient datasets or incomplete datasets, patients still in hospital at the end of the evaluation period and patient deaths during the 6-month post-intervention evaluation period were removed.

The primary outcome measure was the total rate of readmission for all patients pre- and post-intervention. The secondary outcome measure was the total average LoS on the ward for all patients pre- and post-intervention. Means and 95% Confidence Intervals were calculated. For inferential analysis, because the data were not normally distributed (assessed using Shapiro–Wilk’s Test *p* values < 0.05 & inspection of QQ-Plots) and this was a repeated measures design, Wilcoxon Signed-Rank Test (W) was used to examine whether there were any statistically reliable differences in the population pre- and post-intervention (627 pairs). Following visual inspection of the data using histogram plots of the frequency distributions, further analyses were performed to examine the effect of the intervention on the proportion of single count readmissions and three day stays.

Subset analyses of the primary and secondary outcome measures focused on the impact of polypharmacy and readmission rates. Since this variable was also not normally distributed, Spearman’s rank correlation coefficient (ρ) was computed. Subsequently, patients were divided into high and low polypharmacy depending on whether they had more or less than 10 medications at discharge and analysed for pre-and post-intervention admission rates. This classification was based on previous literature identifying patients on  more than 10 medications being strongly associated with adverse drug events [28, 29]. In this case, a non-parametric aligned ranked test (for a two-way factorial design) was performed to examine whether there was an interaction between the intervention and polypharmacy. The threshold for statistical significance across all tests was set at *p* < 0.05. Following data linkage using Microsoft Excel, all preprocessing steps were carried out using the statistical computing environment R v3.4.1 [30].

## Results

**Fig. 1:**
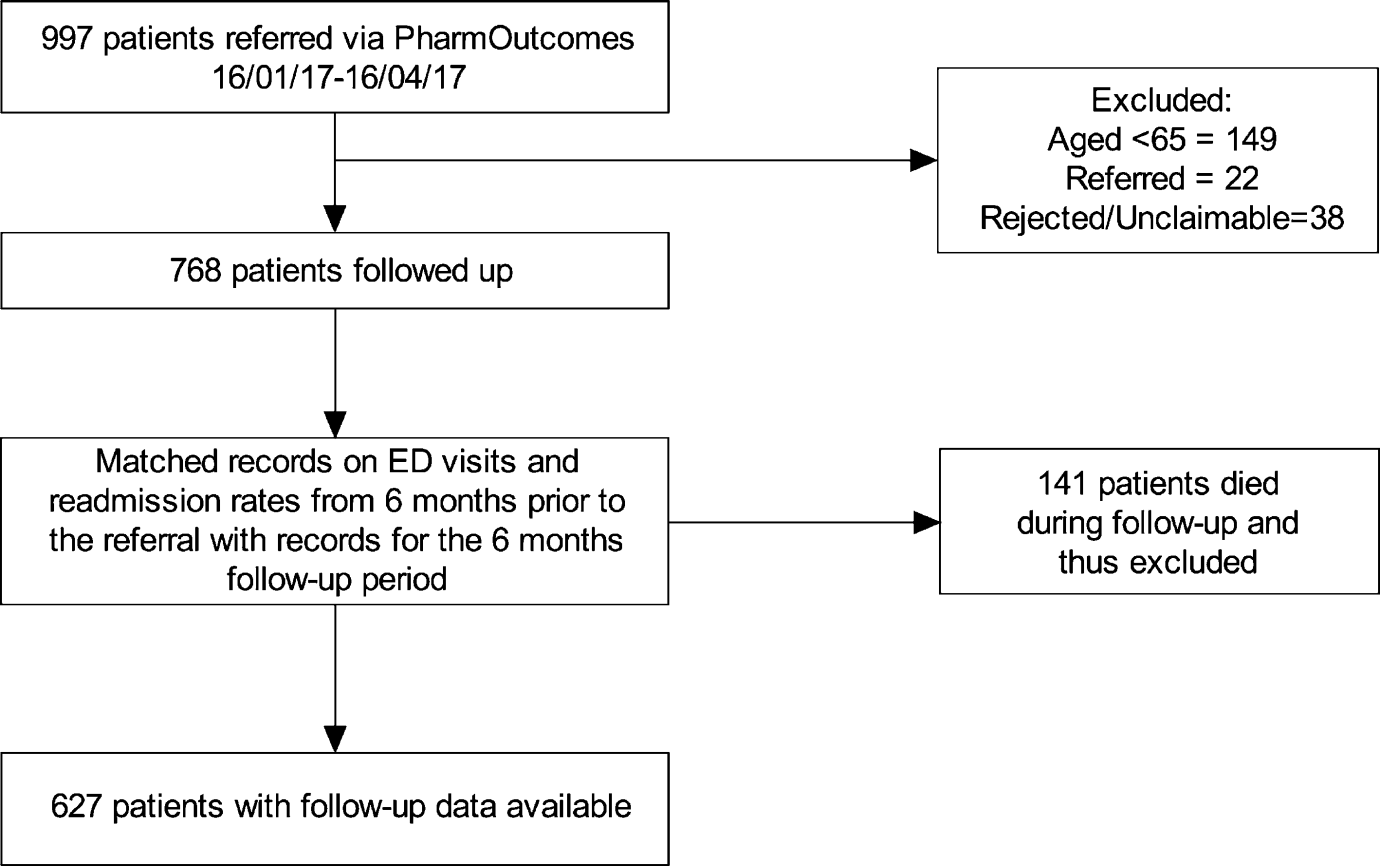
Flow chart detailing the pre-processing steps involved in obtaining the final sample (n = 627) used for statistical analysis from the original dataset comprising 997 patients

A total of 997 patients gave consent for their information to be electronically transferred to their chosen community pharmacy using the CwP intervention during the study period. The focus of this evaluation was the older patient population and, therefore, all patients  under 65 years were removed from the cohort (n = 149). Referrals that were not “completed” or not “actioned” (n = 22) or were deemed “unclaimable” or “rejected” in PharmOutcomes® (n = 38) were also excluded from analysis (see Fig. 1).

The mean age of the final sample was 81.69 years (SD = 7.4 years). Of the referrals made by LTHT, 84% were marked as ‘completed’ by community pharmacies, thus the data examined from the extraction is likely to be a representative reflection of the intervention.

**Fig. 2:**
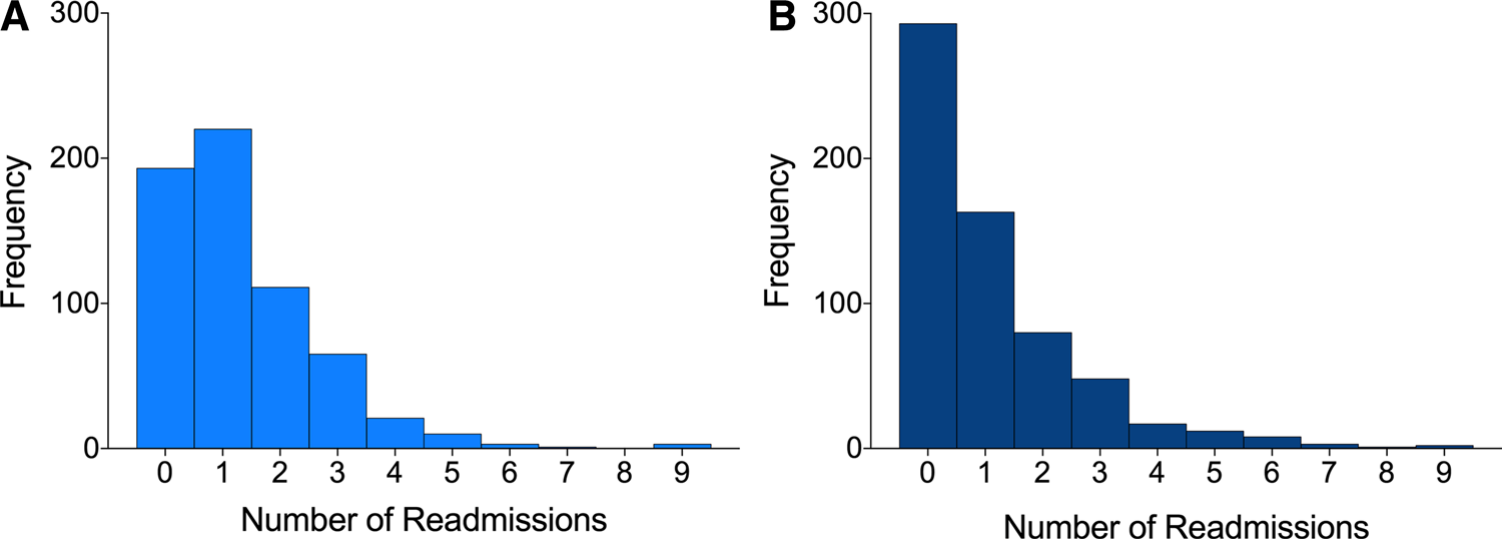
Frequency distribution of the number of total emergency admissions pre-intervention (6 months) (Panel A; light blue) and post-intervention (Panel B; dark blue)

For the primary outcome measure, there was an overall reduction in the frequency of the total number of readmissions post-intervention (n = 690; Mean = 1.1, 95% CI [0.98, 1.22]) relative to admissions prior to the intervention (n = 823; Mean = 1.31, 95% CI [1.21, 1.42]) across all patients in the sample and this difference was statistically significant (W = 54,725; *p* < 0.0001; 200 ties). Visual inspection of the data indicated that the primary impact of the intervention was mediated by increasing the number of zero admissions (see Fig. 2). An overall 16% (n = 133) reduction in readmission was observed.

To explore this more formally, a contingency table with the number of readmissions equal to or more than one was constructed along with the number of zero readmissions post-intervention (see Table 1). Table 1 shows the change in readmissions post-intervention. Here the largest improvements were seen in admission avoidance with the intervention: from the 627 being tracked, 193 had no admissions pre-intervention. Post-intervention, this proportion had increased to 293 patients (a change of 15.95%). Modest improvements were seen in reducing one or more readmissions 6-months post-intervention (from 220 patient pre-referrals to 163; a 9% reduction).

**Table 1:** Proportion of patient readmission rates ≤ 1 & > 1 pre-and post-intervention for the total sample (n = 627)

	Pre-referral admission rates	Post-referral readmission rates	Change
No readmission	193 (30.78%)	293 (46.73%)	+ 100 ( + 15.95%)
Re-admissions equal to 1	220 (35.08%)	163 (26.00%)	− 57 (− 9.00%)
Re-admissions greater than 1	214 (34.13%)	171 (27.27%)	− 43 (− 6.86%)

**Fig. 3:**
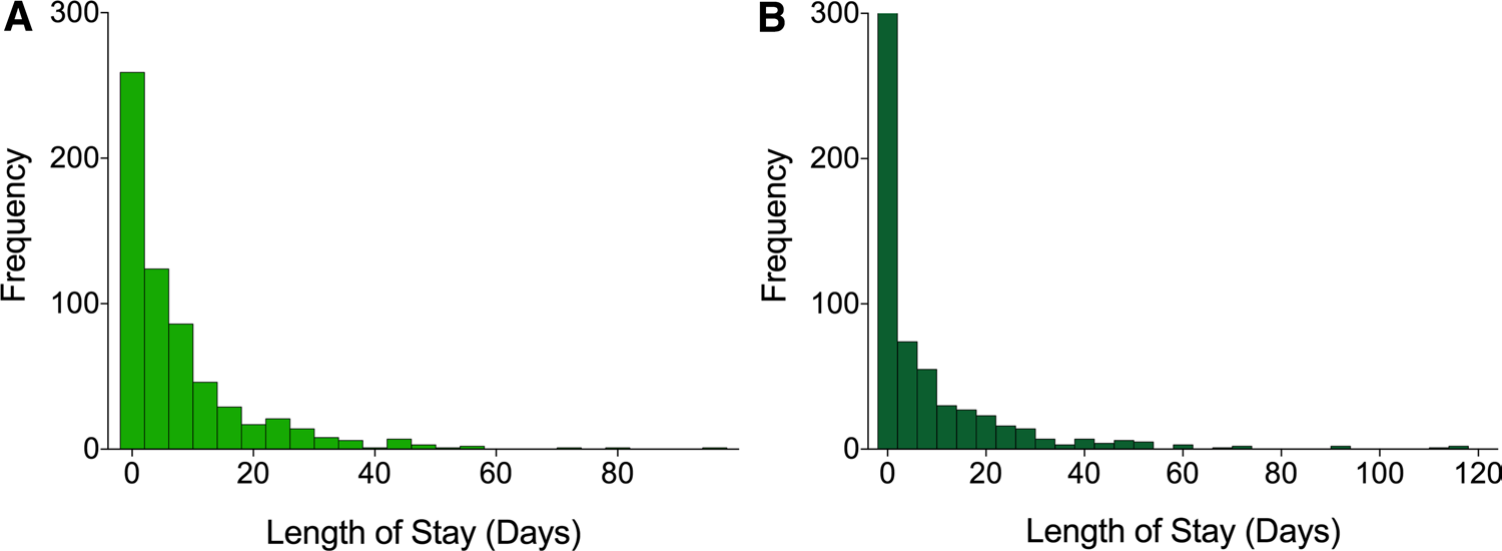
Frequency distribution of the total length of stay on the ward at LTHT pre- (6 months) (Panel A) and post-intervention (Panel B)

For the secondary outcome measure of LoS on the ward, there was a small mean increase post-intervention (Mean = 7.82, 95% CI [6.64, 9.00]) relative to pre-intervention (Mean = 7.40, 95% CI [6.51, 8.29]), but this difference did not reach the statistical significance threshold (W = 63,462, *p* = 0.12; 142 ties). However, an examination of the frequency distribution revealed a similar pattern of results to the primary outcome measure; i.e. any intervention effect was likely to have disproportionately affected those on one tail of the distribution and this effect seemed to be most pronounced in length of ward stays of more than three days (see Fig. 3).

A contingency table was constructed to explore the frequency of LoS duration of ≤ 3 and > 3 days (see Table 2). Statistical analyses revealed that there was a significant difference in the frequency count data (McNemar’s χ^2^ = 13.37, df = 1, *p* < 0.001). In other words, the results suggest that the number of patients spending more than three days in hospital was reduced post-intervention. This was reflected in the fact that out of the 627 patients in our sample, pre-intervention, 237 (52.15%) of the patients spent three days or fewer in hospital, whilst 300 spent greater than three days. After the intervention, the proportion of patients spending three or fewer days in hospital increased to 388 patients, with 239 patients spending greater than three days in hospital (a change of 61 patients and shift of 9.73%).

**Table 2:** Contingency Table showing the proportion of LoS ≤ 3 & > 3 days pre-and post-intervention

	Pre-intervention LoS (%)	Post-intervention LoS (%)	Change
Less than or equal to 3	327 (52.15%)	388 (61.88%)	+ 61 (9.73%)
Greater than 3	300 (47.85%)	239 (38.12%)	− 61 (− 9.73%)

**Fig. 4:**
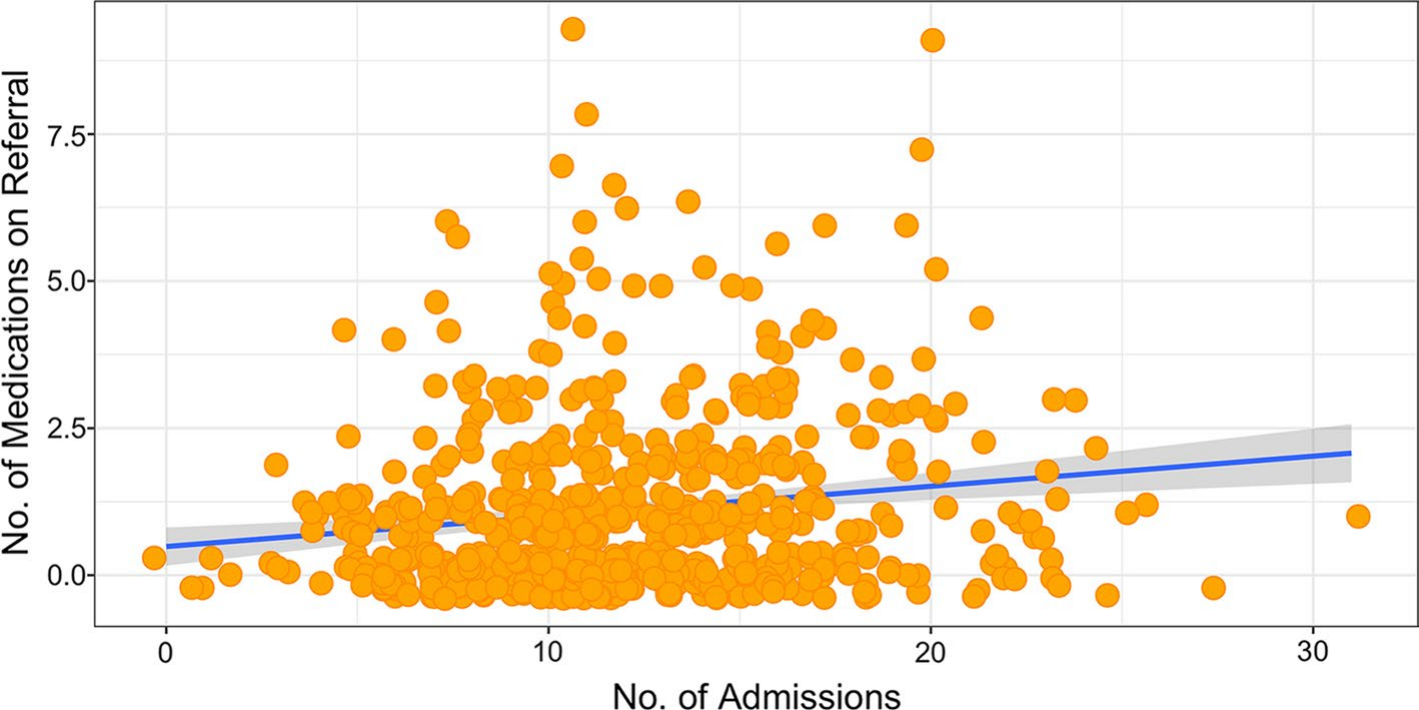
Positive correlation between the number of medications at discharge and the total number of admissions across pre- and post- intervention monitoring periods. Each coloured circle represents an individual patient. The dark grey line indicates best fit and the shaded region around the regression line is the 95% CI

Next, we identified whether the intervention had an asymmetric effect depending on polypharmacy. Preliminary analyses showed a small positive correlation between the number of medications at discharge and the total number of interventions made across the periods examined (ρ = 0.19, *p* < 0.0001; see Fig. 4).

To explore the relationship between the intervention and polypharmacy (mean number of medications at discharge = 11.97, SD = 4.56) data on the primary outcome measure (admissions frequency) was separated based on patients classified as “high” ( > 10 medications on referral) and “low” (< 10 medications on referral) polypharmacy. The difference between number of readmissions pre-and post-intervention tended towards statistical significance in the high polypharmacy group (pre-intervention Mean = 1.4, 95% CI [1.26, 1.55]; post-intervention Mean = 1.25, 95% CI [1.09, 1.41]; *p* = 0.066), and was statistically significant in the low polypharmacy group (pre-intervention Mean = 1.18, 95% CI [1.03, 1.33]; post-intervention Mean = 0.87, 95% CI [0.71, 1.03]; *p* < 0.001). A non-parametric aligned ranked test (for a two-way factorial design) was calculated to explore whether the impact of the intervention was greater in the high polypharmacy or low polypharmacy group, but this test revealed no interaction (*p* = 0.25).

## Discussion

This study evaluated the impact of introducing electronic transfer of a patient’s discharge information to their community pharmacy. The results demonstrated an overall reduction in readmissions after the introduction of the CwP intervention for older people. Subsidiary analyses revealed this effect was most pronounced in preventing readmissions, suggesting this as a potential tool to avoid hospital admissions that may result from post-discharge medication-related problems. Analysis of secondary outcomes revealed that, although there was a trend in a positive direction, there was no statistically reliable reduction in LoS. However, a deeper examination of the data revealed that the intervention seemed to impact on those admissions longer than three days. A similar study [24] with a larger sample size also found significant reduction in LoS for patients readmitted post-intervention, suggesting that the electronic transfer of information interventions may have an impact on LoS. Unfortunately, the reason for readmission was not investigated during the data extraction so these findings must be interpreted with caution. If information detailing the cause of readmission was available, the effect of discrepancies in medicines reconciliation, adverse drug events and other medication-related events on readmission could be sought.

At the outset, it was reasoned that individuals with complex care needs frequently require care in different settings and are particularly vulnerable to experiencing medication problems at each care transition. This vulnerability is further heightened by burden of illness and accompanying polypharmacy [31]. Older people are more likely to experience medication discrepancies after making the transition from hospital to home [11] due to their complex health and social care needs and polypharmacy [32], and therefore have the greatest capacity to benefit from interventions such as CwP.

Recent examination of the literature indicates there is no widely agreed definition for the term polypharmacy. The figure chosen in this study was adopted because taking more than 10 medications has been associated with the greatest risk of adverse drug events [28]. Interestingly in this study, the mean number of medications was 11.97 and the median was calculated to be 11. Although there was no reduction in readmission post-intervention in our high polypharmacy set, our sample was not geared towards finding an effect in polypharmacy. Our next step would be to assess whether a follow up, and what type (such as medication review, provision of advice, medication reconciliation), in such population by the community pharmacist has a significant impact on readmissions.

There are some limitations of this evaluation which would need to be addressed by future work. This study was not a randomised control trial, and, as such, there was no independent arm. Here, all participant pre-intervention data were retrospectively gathered to use as a baseline measure and compared against the same set of patients post-intervention. There are of course several advantages to this quasi-experimental approach e.g. patient demographics are carefully controlled. However, the repeated measures nature also means that the data collected may have been biased by order and time-related effects, having an impact on the internal validity of the study. This makes it difficult to determine whether the CwP intervention was the cause of the reduction in readmissions, or if these patients were likely to have reduced readmissions irrespective of the CwP intervention.

It is also important to consider the extent to which the reported results are generalisable to other settings and patient groups. This phase of the CwP intervention only included patients with a compliance aid and as such, it is possible that the positive effects observed here may be due to the specific patient characteristics of this demographic. Furthermore, 141 patients died during the follow-up period and although these patients were excluded from all steps in the analyses, the intervention may only be appropriate for those at the healthier end of the spectrum. Whether the intervention is also efficacious for older patients with extremely severe health needs, along with those who do not use these aids remains to be seen. We note that in our analyses, we probed whether a proxy of condition complexity (i.e. polypharmacy status) could modulate readmission rates. We did not find an interaction, tentatively suggesting that the effect is not restricted to complex care needs, but further research directly testing this hypothesis on different populations is recommended.

Whilst these results should be treated with a degree of caution given some of the limitations described above, the present analysis indicates that CwP has the potential to improve outcomes for older people and reduce the burden on hospital services by reducing the number of hospital readmissions and relatedly, the number of people who spend more than three days on the wards. Each non-elective admission is estimated to cost NHS hospitals £1,590 whilst an excess bed day in a hospital costs approximately £313 per day [33]. Even modest reductions, when scaled across the NHS could have a substantial economic impact. To provide a rudimentary analysis, in the sample analysed, prior to the intervention, there were a total of 823 admissions and post-intervention, there were 690 admissions. Whilst the 133 fewer admissions are unlikely to all be due to the CwP intervention reported here and a full evaluation of the economic benefit of the intervention is beyond the remit of this study, it can be estimated that £211,470 was saved in this sample post-intervention. Future, rigorous health economics research is necessary to determine the cost-savings of CwP to explore the case for its economic viability.

## Conclusion

The “Connect with Pharmacy” intervention recently adopted by Leeds Teaching Hospitals NHS Trust shows promising evidence, indicating that sharing discharge information with community pharmacies can have a positive impact on older patients’ transfer of care.

## References

[1] Kripalani S, LeFevre F, Phillips CO, Williams MV, Basaviah P, Baker DW (2007). Deficits in communication and information transfer between hospital-based and primary care physicians: implications for patient safety and continuity of care. JAMA.

[2] Witherington EMA, Pirzada OM, Avery AJ (2008). Communication gaps and readmissions to hospital for patients aged 75 years and older: observational study. Qual Saf Health Care..

[3] Royal Pharmaceutical Society. Keeping patients safe when they transfer between care providers—getting the medicines right: Final report 2012. Available from: https://www.rpharms.com/current-campaigns-pdfs/rps-transfer-of-care-final-report.pdf. Accessed 12th Jan 2017.

[4] Dvorak SR, McCoy RA, Voss GD (1998). Continuity of care from acute to ambulatory care setting. Am J Health Syst Pharm..

[5] Chen Y, Brennan N, Magrabi F (2010). Is email an effective method for hospital discharge communication? A randomized controlled trial to examine delivery of computer-generated discharge summaries by email, fax, post and patient hand delivery. Int J Med Inform.

[6] Spencer RA, Spencer SEF, Rodgers S, Campbell SM, Avery AJ (2018). Processing of discharge summaries in general practice: a retrospective record review. Br J Gen Pract.

[7] Avery A, Barber N, Ghaleb M, Dean Franklin B, Armstrong SJ, Crowe S, et al. Investigating the prevalence and causes of prescribing errors in general practice: the PRACtICe Study (PRevalence And Causes of prescrIbing errors in general practiCe) 2012. https://www.gmc-uk.org/-/media/about/investigatingtheprevalenceandcausesofprescribingerrorsingeneralpracticethepracticestudyreoprtmay2012.pdf?la=en&hash=62C1821CA5CCC5A4868B86A83FEDE14283686C29. Accessed 28th Dec 2018.

[8] Parekh N, Ali K, Stevenson JM, Davies JG, Schiff R, Van der Cammen T (2018). Incidence and cost of medication harm in older adults following hospital discharge: a multicentre prospective study in the UK. Br J Clin Pharmacol.

[9] Paulino EI, Bouvy ML, Gastelurrutia MA, Guerreiro M, Buurma H (2004). Drug related problems identified by European community pharmacists in patients discharged from hospital. Pharm World Sci.

[10] Ahmad A, Mast MR, Nijpels G, Elders PJ, Dekker JM, Hugtenburg JG (2014). Identification of drug-related problems of elderly patients discharged from hospital. Patient Prefer Adherence..

[11] Coleman EA, Smith JD, Raha D, Min S (2005). Posthospital medication discrepancies: prevalence and contributing factors. Arch Intern Med.

[12] Himmel W, Kochen M, Sorns U, Hummers-Pradier E (2004). Drug changes at the interface between primary and secondary care. Int J Clin Pharmacol Ther.

[13] Thompson-Moore N, Liebl MG (2012). Health care system vulnerabilities: Understanding the root causes of patient harm. Am J Health Syst Pharm..

[14] Vira T, Colquhoun M, Etchells E (2006). Reconcilable differences: correcting medication errors at hospital admission and discharge. Qual Saf Health Care..

[15] Jasinarachchi KH, Ibrahim IR, Keegan BC, Mathialagan R, McGourty JC, Phillips JR (2009). Delayed transfer of care from NHS secondary care to primary care in England: its determinants, effect on hospital bed days, prevalence of acute medical conditions and deaths during delay, in older adults aged 65 years and over. BMC Geriatr..

[16] Guthrie B, McCowan C, Davey P, Simpson CR, Dreischulte T, Barnett K (2011). High risk prescribing in primary care patients particularly vulnerable to adverse drug events: cross sectional population database analysis in Scottish general practice. BMJ.

[17] Arora VM, Prochaska ML, Farnan JM, D’Arcy M, Schwanz KJ, Vinci LM (2010). Problems after discharge and understanding of communication with their PCPs among hospitalized seniors: a mixed methods study. J Hosp Med..

[18] Driscoll A (2000). Managing post-discharge care at home: an analysis of patients’ and their carers’ perceptions of information received during their stay in hospital. J Adv Nurs.

[19] Knight DA, Thompson D, Mathie E, Dickinson A (2013). ‘Seamless care? Just a list would have helped!’ Older people and their carer’s experiences of support with medication on discharge home from hospital. Health Expect.

[20] Nazar H, Nazar Z, Portlock J, Todd A, Slight SP (2015). A systematic review of the role of community pharmacies in improving the transition from secondary to primary care. Br J Clin Pharmacol.

[21] Hesselink G, Zegers M, Vernooij-Dassen M, Barach P, Kalkman C, Flink M (2014). Improving patient discharge and reducing hospital readmissions by using Intervention Mapping. BMC Health Serv Res..

[22] Neeman M, Dobrinas M, Maurer S, Tagan D, Sautebin A, Blanc A-L (2017). Transition of care: a set of pharmaceutical interventions improves hospital discharge prescriptions from an internal medicine ward. Eur J Intern Med..

[23] Breuker C, Macioce V, Mura T, Audurier Y, Boegner C, Jalabert A (2017). Medication errors at hospital admission and discharge in type 1 and 2 diabetes. Diabet Med.

[24] Nazar H, Brice S, Akhter N, Kasim A, Gunning A, Slight SP (2016). New transfer of care initiative of electronic referral from hospital to community pharmacy in England: a formative service evaluation. BMJ Open..

[25] Elliott RA, Boyd MJ, Salema N-E, Davies J, Barber N, Mehta RL (2016). Supporting adherence for people starting a new medication for a long-term condition through community pharmacies: a pragmatic randomised controlled trial of the New Medicine Service. BMJ Qual Saf..

[26] Trueman P, Lowson K, Blighe A, Meszaros A. Evaluation of the scale, causes and costs of waste medicines. The York Health Economics Consortium and The School of Pharmacy, University of London, 2010.

[27] The Leeds Teaching Hospitals NHS Trust. Connect with Pharmacy 2019. Available from: https://www.leedsth.nhs.uk/a-z-of-services/pharmacy-services/connect-with-pharmacy/. Accessed 25th July 2018

[28] Duerden M, Avery A, Payne R. Polypharmacy and medicines optimisation: making it safe and sound. London: 2013.

[29] Masnoon N, Shakib S, Kalisch-Ellett L, Caughey GE (2017). What is polypharmacy? A systematic review of definitions. BMC Geriatr..

[30] R Development Core Team. R: a language and environment for statistical computing Vienna, Austria: R Foundation for Statistical Computing; 2013. Version 3.4.1. Available from: https://www.r-project.org/. Accessed 2nd Nov 2017.

[31] Slight SP, Howard R, Ghaleb M, Barber N, Franklin BD, Avery AJ (2013). The causes of prescribing errors in English general practices: a qualitative study. Br J Gen Pract..

[32] Describing deprescribing. Drug Therap Bull. 2014;52(3):25. 10.1136/dtb.2014.3.0238 24603125

[33] NHS Improvement. Reference costs 2016/17: highlights, analysis and introduction to the data 2017. https://improvement.nhs.uk/resources/reference-costs/. Accessed 16th June 2018

